# Patient Experience of a Neurology Tele-Follow-Up Program Initiated During the Coronavirus Disease 2019 Pandemic: A Questionnaire-Based Study

**DOI:** 10.1089/tmr.2020.0034

**Published:** 2021-03-01

**Authors:** Mudit Agarwal, Arushi Arushi, Lovedeep Singh Dhingra, Lajjaben Jayeshkumar Patel, Samprati Agrawal, Padma Srivastava, Manjari Tripathi, Achal Srivastava, Rohit Bhatia, Mamta Bhushan Singh, Kameshwar Prasad, Deepti Vibha, Venugopalan Y. Vishnu, Roopa Rajan, Awadh Kishor Pandit, Rajesh Kumar Singh, Anu Gupta, Divya Madathiparambil Radhakrishnan, Animesh Das, Bhargavi Ramanujam, Ayush Agarwal, Arunmozhimaran Elavarasi

**Affiliations:** ^1^ MBBS, All India Institute of Medical Sciences, New Delhi, India.; ^2^ Department of Neurology, All India Institute of Medical Sciences, New Delhi, India.

**Keywords:** COVID-19, patient satisfaction, telemedicine, teleneurology

## Abstract

**Background:** Teleneurology consultations can be highly advantageous since neurological diseases and disabilities often limit patient's access to health care, particularly in a setting where they need to travel long distances for specialty consults. Patient satisfaction is an important outcome assessing success of a telemedicine program.

**Materials and Methods:** A cross-sectional study was conducted to determine satisfaction and perception of patients toward an audio call based teleneurology follow-up initiated during the coronavirus disease 2019 pandemic. Primary outcomes were satisfaction to tele-consult, and proportion of patients preferring telemedicine for future follow-up.

**Results:** A total of 261 patients who received tele-consult were enrolled. Satisfaction was highest for domain technological quality, followed by patient–physician dialogue (PPD) and least to quality of care (QoC). Median (interquartile range) patient satisfaction on a 5-point Likert scale was 4 (3–5). Eighty-five (32.6%; 95% confidence interval 26.9–38.6%) patients preferred telemedicine for future follow-up. Higher overall satisfaction was associated with health condition being stable/better, change in treatment advised on tele-consult, diagnosis not requiring follow-up examination, higher scores on domains QoC and PPD (*p* < 0.05). Future preference for telemedicine was associated with patient him-/herself consulting with doctor, less duration of follow-up, higher overall satisfaction, and higher scores on domain QoC (*p* < 0.05). On thematic analysis, telemedicine was found convenient, reduced expenditure, and had better physician attention; in-person visits were comprehensive, had better patient–physician relationship, and better communication.

**Discussion:** Patient satisfaction was lower in our study than what has been observed earlier, which may be explained by the primitive nature of our platform. Several variables related to the patients' disease process have an effect on patient satisfaction.

**Conclusion:** Development of robust, structured platforms is necessary to fully utilize the potential of telemedicine in developing countries.

## Introduction

Telemedicine, defined as delivery of health care services remotely using information and communication technologies,^1^ is the amalgamation of medicine and the modern-era technology. It has immense potential for chronic neurological disorders since several factors such as impaired mobility, cognitive dysfunction, inability to drive, and dependence on caregivers limit patients' access to health care services, a problem compounded by a paucity of neurologists—close to a billion people in India reside in areas without a practicing neurologist affiliated with a professional society.^2^ However, adoption of telemedicine for chronic neurological disorders has been minimal,^3^ especially in the developing world.^4^

Although there are many studies that have evaluated patient satisfaction to telemedicine services, almost all of them have been performed in developed countries. Furthermore, they suffer from certain methodological flaws, for example, use of nonvalidated instruments to measure satisfaction and small sample size. A recent systematic review found only four studies with a sample size exceeding 100 patients.^5^

Enforcement of social distancing measures to combat the coronavirus disease 2019 (COVID-19) pandemic led to a shutdown of neurology outpatient services at many centers,^6^ bringing telemedicine to the forefront. Patient experience is an important outcome measure to determine the success of any health care service. In this study, we attempt to analyze patient satisfaction, acceptability, and perception toward a teleneurology follow-up program.

### Methodology

Clinical trial number is not applicable for this study. This was a cross-sectional study at a tertiary care hospital in New Delhi, India. The study was approved by the Institutional Review Board. A telemedicine program was initiated by the department of neurology to provide follow-up outpatient consultations to registered patients. All patients were contacted through audio call-based telemedicine by neurology faculty or senior residents. Patients did not have an option of choosing between tele-consult and in-patient consult as the latter was closed in view of the COVID-19 pandemic. Patients were called on the same days on which their regular follow-up visit was scheduled on their registered mobile numbers. Since it was a newly initiated program, most patients were unaware that they would be receiving a call for tele-consult. In cases the patient was unable to communicate, the primary caregiver of the patient was contacted.

**FIG. 1.**
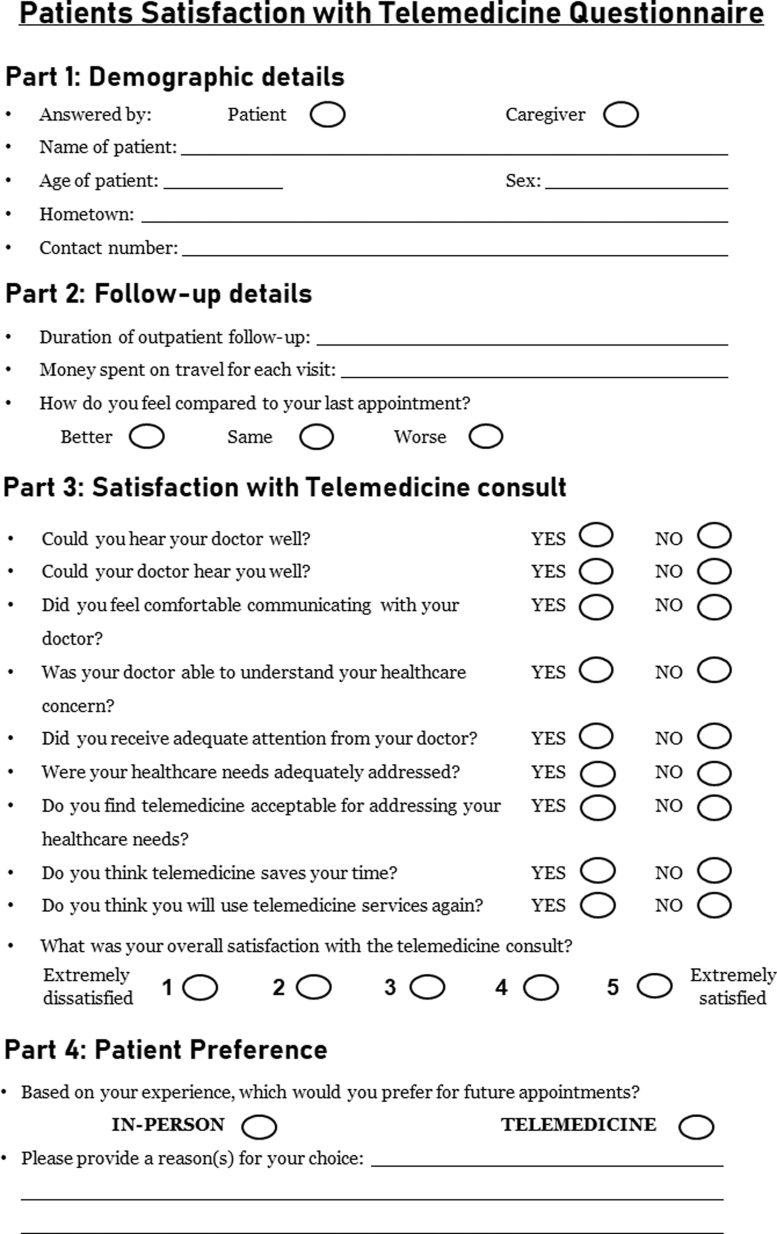
Patient satisfaction with telemedicine questionnaire.

For this study, patients who were contacted for tele-consult between April 18, 2020 and May 20, 2020, who had had at least one in-person neurology follow-up visit previously, were enrolled after verbal informed consent. A questionnaire (Fig. 1) assessing different dimensions of satisfaction^5,7–10^ was created, which included demographic and neurology follow-up details, expenses incurred for in-person visit for transportation and lodging (cost savings with tele-consult), 9 dichotomous scale items to assess satisfaction, and a 5-point Likert scale item for overall satisfaction. Furthermore, to compare tele-consult experience with in-person experience, we also asked for the patients' future preference toward telemedicine or in-person consultations, along with subjective reasons for their choice. This would help in elucidating the aspects in which telemedicine was found to be advantageous and where it was lacking.

As most of the patient community was Hindi speaking, the questionnaire was translated into Hindi by an expert; this version was administered through a phone call by a team of investigators (M.A., L.S.D., S.A., A.A., and L.J.P.) not involved in performing tele-consults. Patients were called twice before they were labeled as not reachable. In cases where the primary caregiver talked to the physician on tele-consult, the survey was administered to the primary caregiver.

### Data analysis

SPSS version 25 was used for statistical analysis. Normality of data was assessed by the Kolmogorov–Smirnov test. Variables were described as median (interquartile range [IQR]) for nonparametric and mean (standard deviation) for parametric variables. Proportions were compared using the Fisher's exact test. Comparison between means was performed using *t*-tests, whereas nonparametric variable were compared using the Mann–Whitney *U*-test (Wilcoxon rank sum test). However, ordinal data were described as median (IQR) and parametric tests were used for analysis, irrespective of normality of the data. This was based on evidence that parametric tests are more robust in yielding unbiased results with ordinal data.^11,12^

Construct validity for the 9-item questionnaire was determined by principal components analysis (PCA) with varimax rotation; number of factors to be extracted was computed by Horn's parallel analysis.^13^ Kuder-Richardson formula 20 was calculated for the factors separately for internal consistency reliability. We generated a composite variable for each factor using weighted averages of the scores obtained on the items constituting the factor, with the weights derived from the factor loadings of the corresponding item obtained from PCA.

The primary outcome measures of the study were overall satisfaction to tele-consult on a 5-point Likert scale and proportion of patients preferring tele-consults for future follow-up visits. We sought to elucidate the factors affecting patient's satisfaction and the reasons behind their preference. Factors affecting overall patients' satisfaction was determined using a multivariable linear regression model adjusted for age, gender, interviewee, hometown, diagnostic category, follow-up duration (log adjusted), patient condition, treatment advised, and the composite variables generated for each factor determined using PCA. Similarly, a logistic regression model was created to determine factors determining patients' preference for future appointments adjusted for the same variables used in the previous model along with overall satisfaction. The cutoff for significance was *p* < 0.05. Furthermore, qualitative analysis of the subjective reasons was done using inductive thematic analysis to capture perceptions that were not covered by the objective part of the questionnaire.

### Data availability

The data sets used and analyzed in this study are available from the corresponding author on reasonable request.

## Results

Between April 18, 2020 and May 20, 2020, a total of 357 patients were contacted for a telemedicine consult. Out of these, 96 were excluded: 73 could not be contacted, informant was unavailable for 19 cases and 4 did not consent.

### Demographic profile and follow-up characteristics

The questionnaire was answered by the individual who consulted with the physician through tele-consult, which was the patient themselves in 122 (46.7%) cases and the primary caregiver in 139 (53.3%) cases (Table 1). Median (IQR) age of the cohort was 32.5 years (22–50). Male preponderance was present (64%). 58.2% of patients were residing in a state different from the hospital.

**Table 1.** Demographic and Follow-Up Characteristics of Study Cohort of 261 Patients

Patient characteristics	*n* (%)
Interviewee	
Self	122 (46.7)
Primary caregiver	139 (53.3)
Gender	
Male	167 (64.0)
Female	94 (36.0)
Hometown	
Same state	109 (41.8)
Different state	152 (58.2)
Diagnosis with examination not necessary	
Epilepsy	103 (39.5)
Cerebrovascular disorder	37 (14.2)
Headache disorders	18 (6.9)
Degenerative peripheral nerve/muscle disorders	14 (5.4)
Neuroinfectious	14 (5.4)
Miscellaneous	11 (4.2)
Dementia	2 (0.8)
Diagnosis with examination necessary	
Neuroimmunological disorders	32 (12.3)
Movement disorders	27 (10.3)
Patient condition	
Better/same	207 (79.3)
Worse	53 (20.3)
Unknown	1 (0.4)
Treatment advised	
Changed	22 (8.4)
No change	239 (91.6)

We categorized the neurological diagnoses based on whether a detailed neurological examination was necessary at every follow-up visit or not. Once a diagnosis has been made, certain groups of patients do not require a comprehensive neurological examination at every follow-up visit; for example, the clinical status of patients with epilepsy^10^ and headache disorders can be assessed adequately by history, stroke patients also may not require a detailed examination as the insult is nonprogressive. In contrast, some diagnoses necessitate a detailed follow-up examination—neuroimmunological disorders such as multiple sclerosis have a variable disease course and relapses can be picked up through findings on examination before they become apparent to the patient, movement disorders require an examination due to the nature of the symptoms. One hundred ninety-nine (76.2%) patients belonged to the diagnostic category not requiring follow-up, whereas 59 (22.8%) belonged to the category requiring a follow-up examination. The miscellaneous category included patients who could not be otherwise categorized such as those with hydrocephalus or noninfectious mass lesions.

Fifty-three (20.3%) patients reported that they felt their condition was worse as compared with their last appointment. Only 22 (8.4%) were advised changes in treatment during telemedicine consultation, rest were advised to continue the same treatment. Median (IQR) follow-up duration since the first visit of the patient to our neurology clinic was 24 months (9.5–60).

### Construct validity

**FIG. 2.**
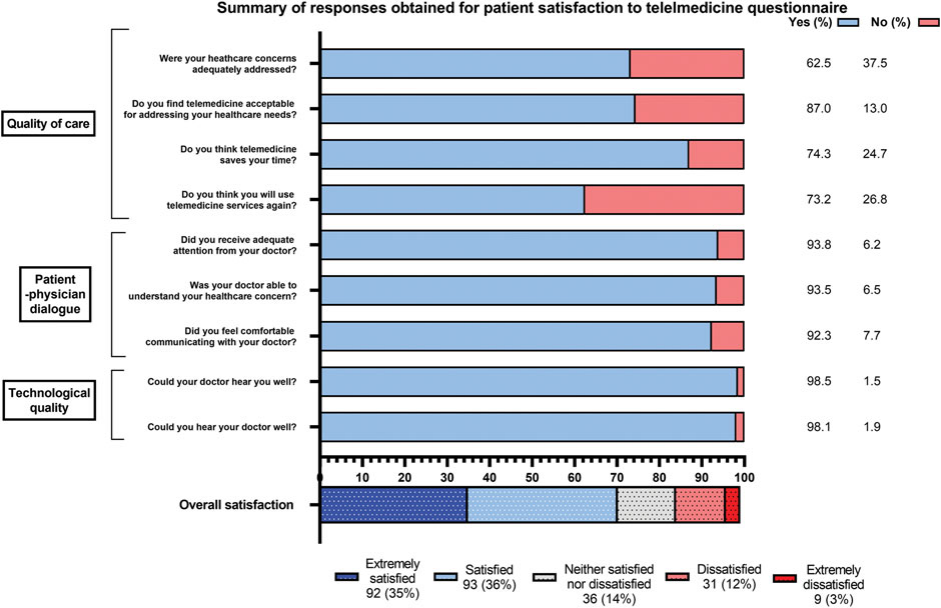
Summary of responses obtained for patient satisfaction to telemedicine questionnaire. The nine questionnaire items were assessed on a dichotomous yes/no scale and were divided into three factors enquiring about separate domains of patient satisfaction–quality of care (4 items), patient–physician dialogue (3 items), and technological quality (2 items). Overall satisfaction was assessed on a 5-point Likert scale.

The questionnaire assessed satisfaction across three distinct constructs/domains of satisfaction as determined by PCA, namely, quality of care (QoC)—4 items, patient–physician dialogue (PPD)—3 items, and technological quality (TQ)—2 items (Fig. 2). These three constructs explained 75.9% of the variance in responses. KR-20 for items within each construct, which was found to be >0.8, signifying that the items within each construct were consistent with each other.

### Cost savings with tele-consult

Median (IQR) cost savings for patients were ₹600/$8.04 (₹250–₹3,000/$3.35–$40.20). Cost savings of patients residing in a different state from that of the hospital (58.2%) were ₹2,000/$26.79 (₹800–₹5,000/$10.72–$66.98) compared with ₹200/$2.68 (₹110–₹400/$1.47–$5.36) for those from the same state. This difference was significant (*p* < 0.001) using the Mann–Whitney *U*-test.

### Satisfaction levels and future preference

Composite scores were generated for the three domains of satisfaction (Table 2). Median (IQR) scores for the domain TQ (maximum score: 0.96) were 0.96 (0.96–0.96); for PPD (maximum score: 0.84) were 0.84 (0.84–0.84) and for QoC (maximum score: 0.79) were 0.79 (0.38–0.79). These scores were significantly different, with least satisfaction to QoC, followed by PPD and TQ (*p* < 0.001). Median (IQR) overall satisfaction level for the tele-consult on a 5-point Likert scale was 4 (3–5; Fig. 2) with 92 (35.2%) answering extremely satisfied, 93 (35.6%) satisfied, 36 (13.8%) neither satisfied nor dissatisfied, whereas only 31 (11.9%) and 9 (3.4%) were dissatisfied and extremely dissatisfied, respectively. Eighty-five patients (32.6%; 95% confidence interval 26.9–38.6%) chose telemedicine as their preferred mode for future follow-up appointments, whereas rest chose in-person visit.

**Table 2.** Characteristics of Study Population and Results of Univariate Analysis for Future Preference of Patients

Variable	Future patient preference		Total (*n* = 261), n (%)	*p*-Value of univariate statistic
	In-person (*n* = 176), *n* (%)	Telemedicine (*n* = 85), *n* (%)		
Interviewee				0.19^*^
Self	77 (63.1)	45 (36.9)	122	
Primary caregiver	99 (71.2)	40 (28.8)	139	
Age (years), median (IQR)	30 (22–53)	35 (22–49)	32.5 (22–50)	0.83^**^
Gender				0.59^*^
Male	115 (68.9)	52 (31.1)	167	
Female	61 (64.9)	33 (35.1)	94	
Hometown				0.28^*^
Same state	78 (71.6)	31 (28.4)	109	
Different state	98 (64.5)	54 (35.5)	152	
Expenditure for in-person visit ($), median (IQR)				0.07^**^
Total	8.04 (2.68–26.80)	13.40 (4.02–40.20)	8.04 (3.35–40.20)	
Same state	2.68 (1.34–5.30)	2.68 (1.34–5.30)	2.68 (1.47–5.36)	
Different state	26.79 (9.93–52.97)	39.73 (13.40–66.98)	26.79 (10.72–66.98)	
Diagnostic category				0.53^*^
Examination not necessary	132 (66.3)	67 (33.7)	199	
Examination necessary	42 (71.2)	17 (28.8)	59	
Unknown			3	
Follow-up duration in months, median (IQR)	24 (9–60)	24 (11–48)	24 (9.5–60)	0.48^**^
Patient condition				<0.001^*^
Better/same	129 (62.3)	78 (37.7)	207	
Worse	47 (88.7)	6 (11.3)	53	
Unknown			1	
Treatment advised				>0.99^*^
Changed	15 (68.2)	7 (31.8)	22	
No change	161 (67.4)	78 (32.6)	239	
Domain: technological quality (maximum, 0.96), median (IQR)	0.96 (0.96–0.96)	0.96 (0.96–0.96)	0.96 (0.96–0.96)	0.03^***^
Domain: quality of care (maximum, 0.79), median (IQR)	0.59 (0.19–0.79)	0.79 (0.79–0.79)	0.79 (0.38–0.79)	<0.001^***^
Domain: patient–physician dialogue (maximum, 0.84), median (IQR)	0.84 (0.84–0.84)	0.84 (0.84–0.84)	0.84 (0.84–0.84)	<0.001^***^
Overall satisfaction (Likert 1–5), median (IQR)	4 (3–4)	5 (4–5)	4 (3–5)	<0.001^***^

Significant *p*-values are highlighted in bold.

^*^ Comparison of proportions across the two groups was performed using the Fisher's exact test.

^**^ Comparison of distribution of nonparametric variables across the two groups was performed using the Mann–Whitney *U*-test.

^***^ Comparison of distribution of ordinal variables across the two groups was performed using the independent samples *t*-test, without assuming equal variances.

IQR, interquartile range.

### Factors affecting overall satisfaction and future preference

We first conducted univariate analyses to compare distribution of variables across the two groups of future patient preference (in-person vs. telemedicine; Table 2). Significant difference was obtained for the variable patient condition at the time of tele-consult; as well as for all three domains TQ, PPD, and QoC and for overall satisfaction.

Multivariate analysis was conducted for 253 cases (8 excluded due to missing data points; Table 2). The linear regression model created with overall satisfaction as the dependent variable elucidated the following factors associated with higher overall satisfaction: Health condition being stable/better (*p* = 0.001), change in treatment advised on tele-consult (*p* = 0.02), diagnosis not requiring detailed follow-up examination (*p* = 0.006), higher score in domain QoC (*p* < 0.001) and domain PPD (*p* < 0.001). Similarly, the logistic regression model created with future patient preference as the dependent variable established that the following factors were associated with a preference for tele-consultation for future appointments: patient themselves consulting with the physician instead of caregiver (*p* = 0.02), less duration of follow-up (*p* = 0.04), higher levels of overall satisfaction (*p* = 0.02), and higher score on domain QoC (*p* < 0.001).

Twenty-two out of 261 cases did not give a subjective reason for their preference. Thus, the responses of 239 participants were included in the qualitative analysis (Table 3); 72 of the 239 participants gave reasons for preferring telemedicine, whereas the rest 167 gave reasons for choosing in-person visits. Inductive thematic analysis of the subjective reasons identified “convenience” and “reduced expenditure” as themes influencing future preference for telemedicine. “Comprehensive care” emerged as the most frequently occurring theme for preferring an in-person consultation, followed by “Better patient-physician relationship” and “Effective communication.”

**Table 3.** Results of Inductive Thematic Analysis of Subjective Reasons Given by 239 Out of the 261 Patients/Caregivers for Future Follow-Up Appointment Preference

Theme	Subtheme	Quotes
Part 1: Preference for telemedicine consultation		
Convenience 70/72 (97.2%)	Saves time	“The same questions are asked in an in-person appointment. Waiting time is at least 3–4 hours at the hospital.”
	47/63 (74.6%)	“It takes me one whole day to travel from my home to the hospital”
	Hassle of travel	“It's a hassle for me to travel with my child.”
	11/63 (17.5%)	“Because of my disease, travelling is very painful”
	Crowding in hospitals 9/63 (14.3%)	“We have a small child and it is very difficult to go to the hospital as it is very crowded.”
		“I sometimes forget to tell everything after… meeting the doctor after long hours of wait; getting to talk to the doctor over the phone …talk in a relaxed manner”
Reduced expenditure 25/72 (34.7%)		“It saves me time and money spent in travelling.”
		“Saves a full day of work.”
Attention paid by physician 6/72 (8.3%)		“The doctor is able to provide more attention than in a busy OPD.”
		“In-person appointment causes a hotchpotch”
Part 2: Preference for in-person consultation		
Comprehensive care 99/167 (59.3%)	Better evaluation	“The doctor is able to examine better, we might not catch signs which a doctor is able to.”
	75/92 (81.5%)	“Since his (patient's) condition is worsening, we would like to show to the doctor in-person so that he can examine him”
	Investigations	“I can't show MRI reports through telemedicine”
	12/92 (13%)	
	Treatment	“I have to get injections of Botox, so can't use telemedicine. The doctor has to examine and then decide on treatment.”
	7/92 (7.6%)	“Mistakes can be made in prescriptions done over calls”
Better patient–physician relationship 40/167 (24.0%)	Personal touch	“I feel more comfortable talking in-person. There is a personal touch.”
	35/40 (87.5%)	“An in-person appointment gives me more mental satisfaction due to the physical presence of a doctor”
	Trust in a physician	“We were not able to consult the doctor that we usually consult.”
	6/40 (15%)	“I need to consult a senior consultant on appointment”
Accessibility of auxiliary services 20/167 (12.0%)	Investigations	“Lab reports are more reliable at the hospital.”
	5/20 (25.0%)	“I need to get various tests done frequently, thus need to consult in-person”
	Medicines	“I buy medicines from the hospital. They aren't available in my hometown, so wasn't able to get a refill”
	8/20 (40.0%)	
	Other departments	“I also have to consult other departments”
	3/20 (15.0%)	
	Avail free services	“I need to get documents signed for refund on drugs, I cannot get that on a call”
	5/20 (25%)	
Effective communication 33/167 (19.8%)		“I am not able to explain problems properly on call.”
		“The doctor couldn't understand me on the phone, since I have a speech problem due to myasthenia”
Miscellaneous 5/167 (3.0%)		“I did not know that I was going to be called, thus was not prepared.”
		“I cannot completely trust a consultation done on call due to fear of fraud”

Responses were classified into appropriate themes and subthemes. Representative responses given by patients are quoted alongside their respective theme/subtheme.

## Discussion

In a developing country with a spread-out health care system, telemedicine can offer a solution to bridge the gap of patient care. Owing to lack of specialist neurological services, patients often have to travel large distances for consults. Although the potential of teleneurology for chronic neurological disorders remains underutilized in India, the COVID-19 pandemic has inadvertently boosted its use.^14^ We aimed to assess the perception and satisfaction of patients toward a recently launched teleneurology follow-up. We included patients who had had a previous in-person follow-up visit, so that patients could measure their tele-consult experience in the context of their usual in-person visit. Our questionnaire assessed patient satisfaction across three constructs—TQ, QoC, and PPD. These constructs fall into broader dimensions of system experience, information sharing, and consumer focus—three areas that are known to influence user satisfaction to telemedicine.^5,8^ Median overall satisfaction observed in our study was 4, whereas other similar studies have reported a higher mean satisfaction of 4.45–4.8 on a 5-point Likert scale (for comparison, mean overall satisfaction of our cohort was 3.87).^5,9,15^ Despite 71% patients being “extremely satisfied” or “satisfied,” only 32.6% patients chose telemedicine for future consults.

The domain *QoC* (encompassing acceptability, addressal of health concerns, saving of time and intention to reuse) influenced overall satisfaction and also strongly influenced future preference. A large study in about 1700 patients also found QoC received on tele-consult to be predictive of preferring telemedicine for future visits.^16^ A successful health intervention should be acceptable and be able to address the patient's problem. Intention to reuse is a surrogate marker of usefulness, whereas telemedicine's ability to save a patient's time and grant convenience is an important contributor to its perceived quality if concerns are addressed as adequately as in an in-person visit. Scores across this domain were significantly lower than the other two domains. This is largely explained by the “primitive” nature of our platform. Patients were not aware that they would be receiving a call, and might not have been prepared for a consult. There might have been difficulties explaining symptoms due to lack of a visual interface, for example, a tremor is better seen than described. Physical examination and reviewing investigations were not possible, thus audio tele-consult could not provide a “comprehensive” evaluation.

The domain *PPD* was also expectedly influencing overall satisfaction—the relative ease and comfort with which a patient can converse and his perception about the extent to which the physician grasping his concerns impacted patient experience. We observed >90% positive responses across this domain.

Nature of the patient's disease and its course also influenced overall satisfaction. New patients presenting to a neurological outpatient usually require a detailed neurological examination that may necessitate an in-person visit.^17^ In contrast, patients with certain established diagnoses not usually requiring a detailed examination at each follow-up visit, such as epilepsy and headache disorders, reported higher satisfaction since for them a telephonic consult would have been as good as an in-person visit. For example, one patient with epilepsy said “the same questions are asked in an in-person appointment…it would be more convenient through telemedicine.” In contrast, certain diagnoses such as movement disorders (due to the nature of their symptoms) and neuroimmunological disorders (due to their variable disease course) require a follow-up neurological examination to assess improvement/worsening of the disease (e.g., assessing tremor and gait in PD, visual acuity in MS)—patients with these conditions reported lower satisfactions since they would have perceived their tele-consult to be incomplete.

Patients who felt they have worsened reported lower satisfaction since they may feel they require an in-depth evaluation; furthermore, the mere physical presence of the physician might instill comfort.^18^ Change in medications was advised in only 22 (8.4%) patients as often detailed examination or review of investigations may be needed. Visual cues such as the patient's body language can influence a physician's decision to prescribe, which cannot be assessed through a telephonic conversation and there may be concerns about the patient not understanding the changes made on a voice call, thus physicians may be wary of modifying treatment on tele-consult. Thus, it is likely that this percentage of treatment change is lower than what would have been if patients had received in-person consults. We observed that treatment change was associated with higher satisfaction. The expectation of a patient to receive a prescription may influence their satisfaction to a consult; patients may correlate a change in treatment to their problems being adequately taken care of, even though it may not be true. Indeed, it has been observed that patients having a chronic disorder and/or those who had seen their physician previously have higher expectations for receiving prescriptions.^19^

We found that only 32.6% patients preferred telemedicine for future visits. Preference for in-person consultation despite high satisfaction to telemedicine has been observed previously in some studies,^20,21^ but the reasons have not been elucidated. We found that when the primary caregiver took the tele-consult, a preference for in-person visits was more likely. This observation demonstrates that caregiver experience was different than in cases the patient themselves consulted with the physician. In neurological disorders, caregivers have a prominent role,^22,23^ for example, about 80% of homecare assistance required by many patients with multiple sclerosis is provided by informal caregivers.^24^ Caregivers may be able to provide a better history for a patient with epilepsy, or they may describe the symptoms better especially in diseases with cognitive impairment, dysarthria, and so on. Caregivers who consulted with the physician may have felt that physician had nil interaction with the patient, and thus have preferred in-person consults. One response was, “tele-consult was good but would like to show in-person as a doctor can pick up things we cannot.” In contrast, a study in patients with MS and other neuroimmunological conditions reported that caregivers may actually prefer telemedicine due to reduced burden of travel, lodging, and so on.^25^

Patients who were on follow-up for longer durations tended to prefer in-person visits—they develop a rapport with their physician and may get accustomed to in-person visits. We found no association between cost incurred for in-person visit and future preference on multivariate analysis.

Thematic analysis generated several insights about the reasons for preference. Telemedicine was convenient, saved both time and money—especially for patients residing in a different state, which comprised 58% of the study population—₹2,000/$26.8, roughly four times the average income of an Indian.^26^ However, in-person appointments provided more comprehensive services and were more “personal” due to interaction with the same physician at every visit. Telemedicine has been observed to be “impersonal” in other studies as well.^27^ Patients requiring multidepartment visits due to other comorbidities or non-neurological manifestations of their diseases; or those receiving services such as free medicines and preferred in-person visits. Communication was also felt to be better in in-person. There may be other barriers that did not come up in our analysis, for example, a resistance to change.^27^ Owing to the foreign nature of telemedicine, patients may be initially reluctant to accept it. Communication with their physician regarding a transition from in-person consults to tele-consults and the advantages offered by telemedicine will likely improve the patients' outlook.

Our study has certain limitations. Our telemedicine program was initiated during the COVID-19 pandemic as a “damage control” measure to mitigate the effect on patients due to shutdown of usual health services. Thus, a simple audio-call platform was used since it could be established with relative ease and could be used to reach all patients, even those without access to smart phones or internet services. However, the lack of a visual interface limits the quality of the interaction between patient and provider. As most structured telemedicine platforms are video based, the generalizability of our results may be limited. All patients could not be reached through the telemedicine platform; this may be partially due to patients being unaware that they would be called for a consult. We were unable to assess all domains of patient satisfaction, for example, concerns about privacy. Certain other variables may also be impacting patient satisfaction, such as their income status, this could not be assessed in the questionnaire. There was a ceiling effect observed among the subdomains of satisfaction in our questionnaire; this may be due to the dichotomous nature of the questionnaire items. We chose a dichotomous scale over a 5-point Likert scale since it is quicker and easier to administer, especially as an interview schedule and can be administered to a wide population with varied cognition and literacy levels without the risk of questionnaire “satisficing.”^28^

However, previous questionnaire-based studies on patient satisfaction have the methodological drawbacks of low sample size and the use of non-validated instruments. In this study, we have validated our questionnaire and have assessed the perception of a large patient population. Thus, despite the limitations, this study has important implications for development of telemedicine platforms, especially in developing countries. Improvements in telemedicine platforms will likely enhance patient experience and increase satisfaction toward telemedicine.

## Conclusion

Although tele-consults offered a convenient and cost-effective alternative for follow-up visits, only 32.6% patients had a future preference for telemedicine. Improvements in telemedicine platforms will likely enhance patient experience and increase satisfaction toward telemedicine. The results of our study should be viewed as an encouragement to adopt robust telemedicine platforms (including video features, electronic prescriptions, and a system for patients to scan reports) for routine follow-up visits in developing countries. A program integrating tele-consults along with in-person visits would offer patients the best of both worlds.

## Notes

**Cite this article as:** Agarwal M, Arushi A, Dhingra LS, Patel LJ, Agrawal S, Srivastava P, Tripathi M, Srivastava A, Bhatia R, Singh MB, Prasad K, Vibha D, Vishnu VY, Rajan R, Pandit AK, Singh RK, Gupta A, Radhakrishnan DM, Das A, Ramanujam B, Agarwal A, Elavarasi A (2021) Patient experience of a neurology tele-follow-up program initiated during the coronavirus disease 2019 pandemic: a questionnaire-based study, *Telemedicine Reports* 2:1, 88–96, DOI: 10.1089/tmr.2020.0034.
